# Mapping the Lipids of Skin Sebaceous Glands and Hair Follicles by High Spatial Resolution MALDI Imaging Mass Spectrometry

**DOI:** 10.3390/ph15040411

**Published:** 2022-03-28

**Authors:** Fang Xie, Mark Reid Groseclose, Sara Tortorella, Gabriele Cruciani, Stephen Castellino

**Affiliations:** 1 Bioimaging Department, GlaxoSmithKline, Collegeville, PA 19426, USA; fang.x.xie@gsk.com (F.X.); reid.x.groseclose@gsk.com (M.R.G.); 2 Molecular Horizon s.r.l., Via Montelino 30, 06084 Bettona, Italy; sara@molhorizon.it; 3 Department of Chemistry, Biology and Biotechnology, University of Perugia, Via Elce di Sotto 8, 06123 Perugia, Italy; gabriele.cruciani@unipg.it; 4 Xenovista LLC, Chapel Hill, NC 27516, USA

**Keywords:** MALDI imaging mass spectrometry, sebaceous glands, hair follicles

## Abstract

Matrix-assisted laser desorption/ionization (MALDI) imaging mass spectrometry (IMS) is a technology that utilizes the high sensitivity and specificity of mass spectrometry, combined with a high spatial resolution to characterize the molecular species present in skin tissue. In this article, we use MALDI IMS to map specific lipids characteristic of two important skin appendages in minipig skin: the sebaceous glands and hair follicles. A set of specific lipid markers linked to the synthesis of sebum, stages of sebum production, and the secretion of sebum for two different sebaceous gland subzones, the peripheral and central necrotic, were identified. Furthermore, biochemical pathway analysis of the identified markers provides potential drug-targeting strategies to reduce sebum overproduction in pathological conditions. In addition, specific lipid markers characteristic of the different layers in the hair follicle bulge area, including the outer root sheath, the inner root sheath, and the medulla that are associated with the growth cycles of the hair, were determined. This research highlights the ability of MALDI IMS to link a molecular distribution not only to the morphological features in skin tissue but to the physiological state as well. Thus, this platform can provide a basis for the investigation of biochemical pathways as well as the mechanisms of disease and pharmacology in the skin, which will ultimately be critical for drug discovery and the development of dermatology-related illnesses.

## 1. Introduction

Imaging mass spectrometry (IMS) has provided a “molecular” basis for tissue histology [1,2,3]. Through co-registration of molecular ion images and histological stained tissue images, the biological knowledge gained by the careful examination of tissue morphology can be integrated with molecular species that can be linked to biochemical pathways. Thus, by combining these two imaging modalities, a more holistic view of biology and chemistry emerges. The highly sensitive, specific, and label-free study design of IMS combined with high spatial resolution make this imaging modality a valuable tool in the study of disease pathogenesis and pharmacology.

In this study, we employed matrix-assisted laser desorption–ionization (MALDI) IMS to acquire high lateral resolution (10 µm) images of dorsal skin from a Göttingen minipig, an important preclinical species for topical drug development. Multivariate statistical analysis was adopted for data mining, with the goal of using a series of 2D tissue section planes to capture the molecular basis for the structural elements of tissue morphology and to reconstruct the physiological process of sebum production.

We targeted sebaceous glands and hair follicles, two important skin appendageal structures, because of their relevance to skin diseases and the delivery of topically applied medicines. Sebaceous glands are responsible for the synthesis and secretion of sebum to protect against microbial infection and to prevent water loss [4,5]. Overproduction of sebum is linked to the pathogenesis of acne vulgaris [6,7]. Inhibition of de novo lipid synthesis, such as triglycerides, to reduce sebum production has been a favorable strategy for the treatment of acne [8]. Hair follicles are important for warmth function in mammals and can also serve as transappendageal routes for delivering topically applied medicines past the stratum corneum barrier to therapeutic targets localized in the epidermis and dermis, including the sebaceous gland itself [9,10]. In terms of molecular species identification, we specifically focused on lipids, given their critical roles in sebaceous gland functions. For hair follicles, although the role of lipids has not been extensively studied, it has been recognized that the perturbation of fundamental lipid pathways can cause severe hair follicle injury and consequently impact the normal function of sebum delivery or the transappendageal delivery of topical medicines [11].

A limited number of studies have attempted to image the lipid signals in sebaceous glands and hair follicles. In one study, Coherent anti-stokes Raman scattering (CARS) microscopy was employed to image the dynamic lipid changes in mouse ear sebaceous glands during the holocrine secretion process. These CARS experiments demonstrated that the weakest lipid signal was associated with the lipid-poor progenitor sebocytes residing along the periphery of the sebaceous gland. The lipid signals from sebocytes in the interior portions of the gland were stronger, while the strongest lipid signal was mapped to the ductal outlet [12]. Another study utilizing volumetric multispectral optoacoustic tomography (vMSOT) was carried out recently to characterize hair follicles and surrounding structures [13]. An advantage of vMSOT is the noninvasive volume measurement of deep tissue chromophores including lipids. In this study, the major and minor axes of an ellipsoid, which was surrounding a hair follicle and displayed a strong lipid signal, were measured, and the overall lipid volume was estimated to be 153 µL [13]. While these in vivo imaging techniques provide some insights into the structure and function of the sebaceous gland and hair follicles, they are not able to relate molecular specificity to the structural features or the biochemical processes. From this perspective, our study stands out, as it exemplifies that IMS can provide a complementary imaging modality that can bridge biology and chemistry to realize “molecular histology”.

## 2. Results and Discussion

### 2.1. Identification of Lipids Associated with the Physiological Stages of the Sebaceous Glands (SG)

**Figure 1:**
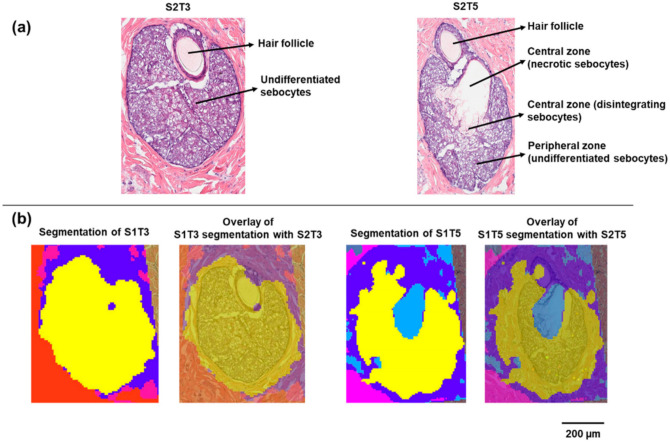
Histology and segmentation analysis of sebaceous glands on minipig skin sections: (**a**) H&E staining of the sebaceous glands on sections S2T3 and S2T5; (**b**) segmentation clustering of the sebaceous glands on S1T3 and S1T5.

Two sets of serial skin sections were collected for MALDI IMS and H&E staining. On each slide, four sections were mounted with known spatial distances, as shown in Figure S1, Supplementary Materials. In the CARS microscopy study of Jung et al., the dynamic process of sebocyte migration from the periphery toward the gland duct was accompanied by the change in lipid signals, which was attributed to the degradation of sebocytes during holocrine secretion [12]. This process can also be reconstructed, at least partially, from a series of 2D serial histological sections. As shown in Figure 1a, the SG captured in S2T3 consisted entirely of undifferentiated sebocytes. These small rounded sebocytes packed with lipid-filled vacuoles are characteristic of the early stage of sebum development. As shown in section S2T5, which is approximately 90 µm away from S2T3, differentiated sebocytes progressively migrated toward the SG center. A necrotic region containing ruptured sebocytes that released their lipid contents can also be observed. In this section, undifferentiated sebocytes in the peripheral zone remained intact.

MALDI IMS was performed on serial sections (S1) adjacent to sections for H&E staining (S2) with 10 µm pixel dimensions. Unsupervised segmentation analysis was performed as an initial exploratory data analysis step to identify molecular species that provide differentiation between various tissue features. In this approach, pixels with similar mass spectra, as determined by the clustering algorithm, were grouped into blob masks. A bisecting k-means segmentation algorithm was selected in this study because (1) k-means and bisecting k-means (a modification over basic k-means) are well known for their compute-time efficiency, compared with the time-consuming hierarchical clustering [14,15] and (2) bisecting k-means carries the merits of k-means and offers additional advantages such as greater exactitude, no generation of empty clusters, and improved efficiency [15]. As shown in Figure 1b, on section S1T3, the entire SG was assigned to one cluster (yellow), compared with the other tissue areas (red and blue blob masks). In contrast, the SG on section S1T5 was differentiated into two-pixel clusters based on the MALDI IMS data. These two clusters correspond to the peripheral zone of the SG (yellow) containing undifferentiated sebocytes and the central zone (light blue) containing the disintegrating and necrotic sebocytes with released contents, respectively, which can be further visualized by overlaying the segmentation blob masks with the serial H&E images (Figure 1b).

**Figure 2:**
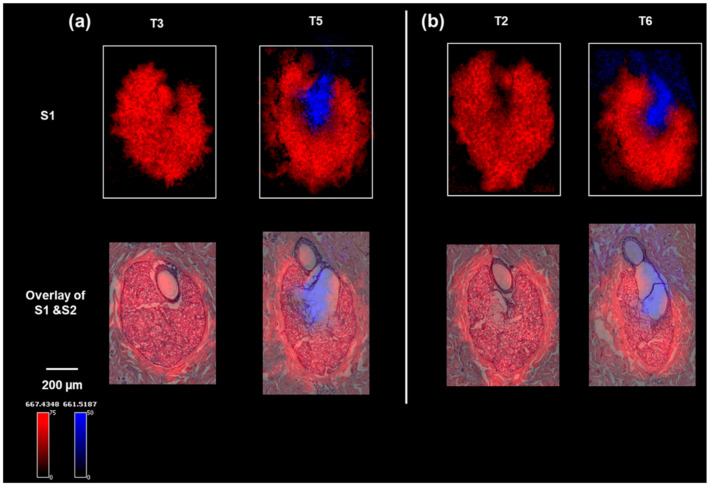
Ion images of sebaceous glands on minipig skin sections: (**a**) ion images of *m*/*z* 667.4348 (a peripheral zone marker) and *m*/*z* 661.5175 (a central zone marker) on the sebaceous glands of S1T3 and S1T5, and overlaid with H&E staining of the sebaceous glands on section S2T3 and S2T5; (**b**) ion images of *m*/*z* 667.4348 (a peripheral zone marker) and *m*/*z* 661.5187 (a central zone marker) on the serial sections of S1T2 and S1T6, and overlaid with H&E staining of the sebaceous glands on section S2T2 and S2T6.

To identify the lipids that were associated with the two specific physiological stages of sebum production in the sebaceous glands, the MALDI IMS data were clustered into regions of interest (ROIs) based on the segmentation results, after which a correlation analysis was performed on the data from S1T5, which contained both SG zones, to find molecular ions (*m*/*z* values) co-localized to the same ROI. Two representative lipid-ion images (which can be definitively identified in the parallel LC–MS–MS analysis) for S1T3 and S1T5 are shown in Figure 2a. The distributions of these two ions are highly consistent with the two physiological zones identified on the H&E. The small differences in ion distribution compared with the H&E staining can be explained by the histological variations between S1 and S2 serial sections, as shown by the optical images of S1 (Figure S2, Supplementary Materials). These two lipid markers were also shown to map with high specificity to the same histological structures (the peripheral zone or central zone) on other serial tissue sections, as shown in Figure 2b, further validating the specificity of the markers. Table S1 ( Supplementary Materials) lists the identified or annotated lipids based on LC–MS–MS analysis of serial section homogenates. Combining the co-localization analysis and ion maps, nine lipids were shown to be markers for the peripheral zone and eight lipids were associated with the central zone. These markers covered six classes of lipids. In S1T2 and S1T3 (Figure 2), the absence of SG central zones was accompanied by a lack of diglyceride (36:1) (DG 36:1; *m*/*z* (M + K)+ 661.5187) detection. In S1T5 and S1T6, the DG (36:1) ion was detected in the central zone and above, which correlates with the release of the sebum from the sebaceous glands into the follicle canal (Figure 2). Notably, DGs were detected as (M + K) + by MALDI IMS but as (M + NH4) + by LC–MS–MS.

**Figure 3:**
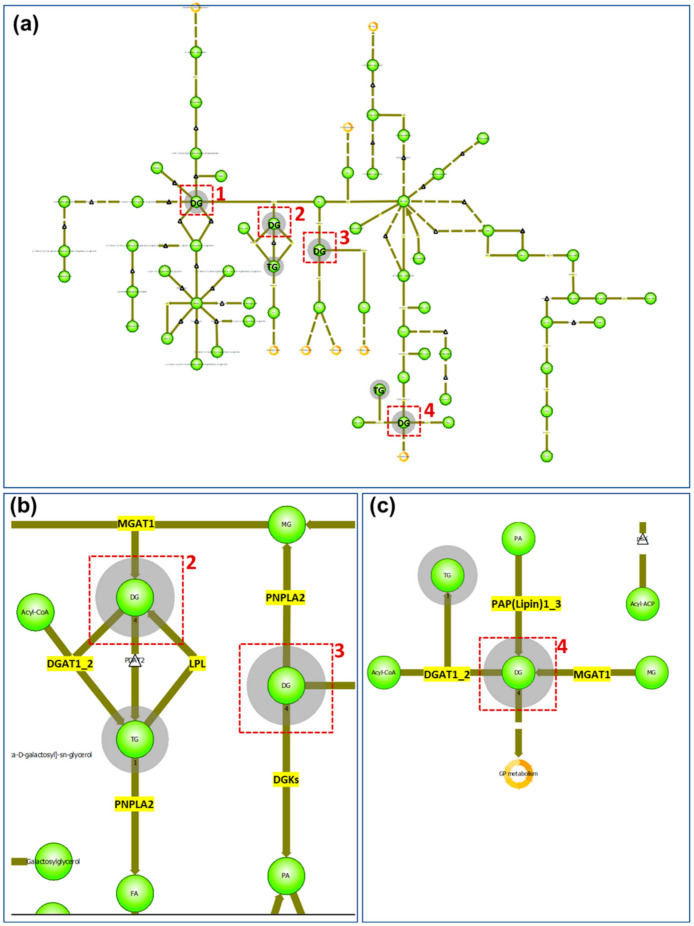
Glycerolipids pathway analysis of SG central zone markers: (**a**) global view of glycerolipids pathway. Red boxes indicate the nodes of DGs and the grey shadowed circles indicate the lipid classes detected from the imported list of compounds; (**b**) enlarged view of nodes 2 and 3 from (**a**). Enzymes existing in humans are highlighted in yellow; (**c**) enlarged view of node 4 from (**a**) with enzymes existing in humans highlighted in yellow.

Most of the lipids identified or annotated that were specifically associated with the peripheral zone of the sebaceous gland are phospholipids. This is not unexpected. as phospholipids are among the major components of all cell membranes, and the sebocytes in the peripheral zone are not fully mature and have intact cell membranes. In the central zone, where sebum is excreted from the sebocytes, four out of the eight lipid markers are DGs in addition to two triglyceride (TG) species. In a MALDI IMS study, where lipids excreted by normal skin were compared with those excreted to the skin surface by SGs derived from cultured epidermal stem cells [16], it was shown that TGs and DGs are among the major components common in both cases [16]. Furthermore, in a recently published study, DGs were found to be more abundant in sebum from adolescents affected with acne than other lipid species [17]. Another study that employed time-of-flight–secondary ion mass spectrometry (TOF–SIMS) to image human skin found DG signals strongly localized to the skin surface, hypodermis, and specific structures deep into the dermis that appeared to be exocrine glands [18]. A drawback of TOF–SIMS analysis is that DG signals could be attributed to TG species, as TGs are extensively fragmented in TOF-SIMS producing DG ions [18]. In contrast, DGs and TGs are readily observed by MALDI MS and detected as Na^+^ or K^+^ adducts in positive ion mode [19]. It is established that TGs are synthesized in the sebocytes as a major component of sebum [20], and as the sebum flows outward through the follicle canal, a variable proportion of the TGs can undergo lipase-mediated hydrolysis forming both fatty acids and DGs [21]. Our data show that DGs are a main component in the secreted sebum from the mature sebocyte, which is consistent with the reported hydrolysis reaction of TGs in the maturation of sebocytes from the progenitor cells. In addition, when the identified/annotated markers of the SG central zone are imported into the pathway analysis of LipostarMSI, focusing on the glycerolipids (because six out of the eight markers belong to the glycerolipid class), four nodes were detected involving DG/TG metabolism (Figure 3a). In node 1, TGs are not involved, and enzymes participating in DG metabolism do not exist in humans; therefore, we focused on nodes 2, 3, and 4 to elucidate the metabolism of DGs and TGs. In nodes 2 and 3 (Figure 3b), besides the hydrolysis of TGs catalyzed by LPL, DGs can be formed from monoglycerides catalyzed by MGAT1. DGs can also be converted back to monoglycerides through the PNPLA2-catalyzing pathway. PNPLA1 also catalyzes the degradation of TGs to generate DGs. Another pathway of DGs catabolism is through the enzyme DGKs to form phosphatidic acids. In node 4 (Figure 3c), DGs can be synthesized from monoacylglyceride catalyzed by PAP(lipin)1–3. A summary of the enzymes involved in the formation and catabolism of DGs and TGs from the pathway analysis is shown in Table S2 (Supplementary Materials). Considering that TGs are a major component of sebum, and DGAT-catalyzed synthesis is the only pathway to the final formation of TGs, it follows that inhibition of DGAT is a favorable drug targeting strategy to combat overproduction of sebum in acne vulgaris [22]. Our findings in this study provide potential alternatives to reduce sebum secretion, including inhibition of TG hydrolysis catalyzed by lipoprotein lipase, inhibition of DG synthesis from monoacylglyceride through phosphatidate phosphatase, or induction of kinase-directed DG degradation.

**Figure 4:**
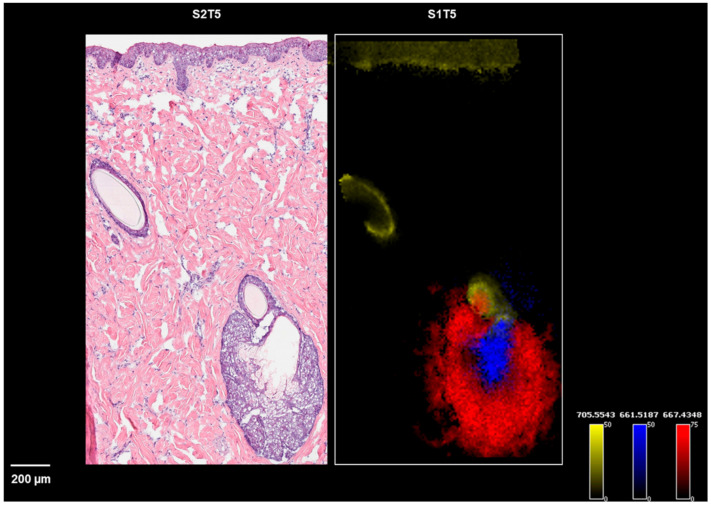
Ion images of *m*/*z* 667.4348 (a peripheral zone marker), *m*/*z* 661.5187 (a central zone marker), and *m*/*z* 705.5543 (a hair follicle and epidermis marker) on sections of S1T5, along with the H&E staining of S2T5.

Notably, a few diacylglycerophosphocholines (PCs), along with a ceramide phosphoethanolamine (Table S3, Supplementary Materials), were identified or annotated to be specifically associated with the stratum corneum and the two hair follicles shown in Figure 4. Among them, ceramide phosphoethanolamines (40:1) and PC (40:1) were detected in two ion forms, indicating their dominant abundance in the stratum corneum. In a recent study published by Hart et al., the MALDI images acquired from ex vivo human skin also showed the dominant abundance of ceramide phosphate in the epidermis compared with dermis [23].

### 2.2. Identification of Lipids Associated with the Specific Regions of the Hair Follicles

**Figure 5:**
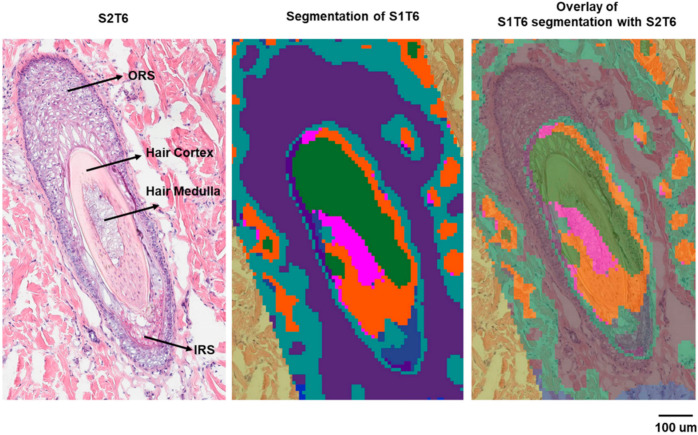
Histology and segmentation analysis of hair follicle-3.

A similar approach was used to correlate the biological features of the hair follicle with specific molecular species. The hair follicle is a complex structure composed of multiple cell types and different compartments [24]. It can be divided into several regions: (1) the infundibulum, which begins at the entrance of the sebaceous gland duct and continues to the opening on the epidermis; (2) the isthmus, the region below the sebaceous duct; (3) the bulge, the area located where the arrector pili muscle is in contact with the hair follicle; (4) the hair bulb embedded in the dermal adipose tissue [25]. Each region displays characteristic histology and represents a certain stage of the hair growth cycle. On the serial sections collected in this study for histology, three hair follicles were identified by H&E staining (Figure S3, Supplementary Materials). The hair follicle we characterized (hair follicle-3) is deeply rooted in the dermis with various layers displayed, including the bulge region of the hair follicle. The bulge region is important for regulatory processes during hair growth [25] because of the stem cells residing there [26]. The segmentation analysis showed that ions detected by the MALDI IMS can be clustered in alignment with the histological layers of this hair follicle (Figure 5).

**Figure 6:**
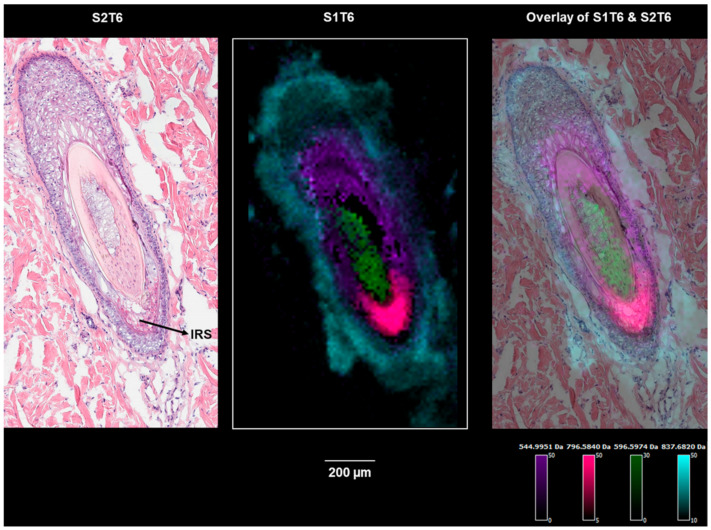
Ion images of *m*/*z* 544.9959 (a cortex marker), *m*/*z* 837.6818 (an outer root sheath marker), *m*/*z* 596.5974 (a medulla marker), and *m*/*z* 796.5840 (an inner root sheath marker) on section S1T6 showing hair follicle-3 (the bulge region).

The correlation analysis was performed to find the ions localized within the regions that are defined by the segmentation result and the corresponding histological layers of the hair follicle. Table S4 (Supplementary Materials) lists the identified/annotated lipids that are associated with the outer root sheath (ORS), inner root sheath (IRS), and the hair medulla, respectively. No lipids were definitively identified for the IRS or medulla, presumably because of the relatively smaller areas they occupy, and therefore, the localized lipids were more extensively diluted in the homogenate for LC–MS analysis. In future investigations, a droplet-based liquid microjunction surface sampling technique will be employed to further identify the highly localized markers. In Figure 6, exemplary ion images of the identified/annotated lipids are displayed. A nonlipid molecule (the purple ion image in Figure 6) specifically associated with the keratinized cortex can also be isolated and is consistent with the generally proteinaceous character of the cortex [27]. Supplemental Figure S4 (Supplementary Materials) displays the same panel of ions from Figure 5 on a serial section, which is approximately 100 µm away. In this section (Figure S4), the IRS located above the medulla is associated with PC (37:1) (*m*/*z* 840.5879, red). The absence of the cortex in this section (S1T3) was accompanied by the lack of the corresponding nonlipid ion marker (*m*/*z* 544.9959). It is interesting that the lipids in Table S2, which are associated with hair follicle-1 and follicle-2, are not expressed in follicle-3 (Figure S4), further indicating the different physiological phases of follicle-3, compared with the more differentiated follicle-1 and follicle-2. On the other hand, the lipids associated with the ORS of follicle-3, including a series of sphingomyelins and PCs, are also detected in the epidermis. The lower bulge region represented by follicle-3 contains stem cells that can differentiate into cells that form the hair shaft during the renewal process [26]. This renewal differentiation consists of cyclic phases of telogen, anagen, and catagen [28], and is orchestrated by matrix progenitors. However, the molecular and temporal signaling pathways remain unclear [29]. The lipids associated with specific hair follicle regions can provide insights into future signaling pathway investigations.

In the 3D in vivo images of hair follicles obtained by vMSOT, the volume of lipids surrounding a superficial hair follicle in the scalp was estimated and compared with the lipids dispersed from a deeper hair follicle on the hand [13]. The lipid signal near the superficial follicle was strong and concentrated in contrast to the dispersed signals surrounding the deep hairs. The authors assumed that the lipid signal close to the skin surface is from the sebaceous glands surrounding the hair shaft, whereas the deep lipids may correlate to the subcutaneous fat [13]. However, in vivo imaging is incapable of unveiling the molecular identity of the lipids; consequently, interrogation and understanding of the associated biochemical pathways are not possible. In our results, we can clearly see that the lipids identified as ORS markers are also expressed in the epidermis (Figure S5, Supplementary Materials). This agrees with the developmental biology of skin, as ORS cells can differentiate into the epidermis [30]. The ORS also exists in the other two hair follicles. In contrast, the IRS and the hair medulla are not identified in hair follicle-1 and hair follicle-2, and consequently, their lipid markers; a dialkylglycerophosphoglycerols, two diacylglycerophosphocholines (37:4 and 37:1), a dihydroceramide (38:0), and a 1-alkyl,2-acylglycerophosphate (34:1) were minimally detected in these two follicles, as shown in Figure S5.

## 3. Materials and Methods

### 3.1. Materials and Reagents

HPLC grade methanol, ethanol, and trifluoroacetic acid (TFA) were purchased from Fischer Scientific (Pittsburgh, PA, USA), while 2,5-dihydroxybenzoic acid (DHB, purity 98%) was purchased from Sigma Aldrich (St. Louis, MO, USA) and purified by recrystallization. Andwin Scientific Tissue-Tek Optimum Cutting Temperature (OCT) embedding media was purchased from Fischer Scientific (Pittsburgh, PA, USA).

### 3.2. Tissue Sample Preparation

Minipig (nontreated) skin samples were selected from a pharmacokinetic study conducted by the contract research organization (Envigo CRS, Inc., Eastmillstone, NJ, USA). The samples were stored at −80°C before sectioning. OCT was used to mount the tissue for cryosectioning. The skin samples were sectioned at a thickness of 6 µm by a Leica Cryostat (Leica Biosystems Inc., Buffalo Grove, IL, USA) at −20 to −25°C.

### 3.3. MALDI Imaging Mass Spectrometry

The matrix DHB was sublimed onto skin tissue sections for MALDI IMS by using a custom-built sublimation apparatus. Under stable vacuum (~200 to 500 × 10^−3^ Torr) and temperature (130 °C), 30 mg DHB was sublimed to the section. All MALDI IMS was performed using a Solarix 7T Fourier transform ion cyclotron resonance mass spectrometer (Bruker Daltonics, Billerica, MA, USA) operating at 1 kHz providing a laser spot diameter down to ~10 μm for the “minimum” focus at the laser power of 30% with 50 laser shots. Mass spectra were acquired in full scan mode (*m*/*z* 200–1500).

### 3.4. LC–MS Analysis

Additional tissue sections (~100) serial to those collected for MALDI IMS were collected in preweighed vials, and ~0.5 mL of water was added prior to homogenization. The tissue was homogenized at room temperature using an Omni Bead-24 Ruptor (Omni Inc., NW Kennesaw, GA, USA) for 15 s at 7.1 M/s, followed by 10 s at 5.8 M/s. The homogenates were extracted following a published method for lipids analysis [31]. The supernatant was analyzed using an Agilent 1200 HPLC, coupled with a Thermo LTQ-Orbitrap operated in positive ion mode. The following parameters were used: source temperature 300 °C; ionization voltage 4.5 kV; mass range 200–900; normalized collision energy (NCE) 35. The LC method was based on a published method [32]. Briefly, the chromatographic separation was performed using an Agilent 1260 system (Agilent Technologies, Santa Clara, CA, USA) equipped with a Supelco Discovery HS F5 column (Sigma-Aldrich, St. Louis, MO, USA). A binary gradient was employed: Solvent A consisted of water–methanol (1/1, *v*/*v*), and solvent B was 2-propanol. Both solvents contained phosphoric acid (8 µM), ammonium acetate (10 mM), and formic acid (0.1 vol%). The linear gradient started at 45% solvent B and increased to 100% solvent B within 21 min. Solvent B percentage was kept at this level for 5 min. Starting conditions were achieved in 0.5 min and the column was re-equilibrated for 3.5 min, resulting in a total HPLC run time of 30 min.

### 3.5. IMS and LC–MS Data Processing and Multivariate Statistical Analysis

The peak lists of IMS data (sqlite files) were imported to an in-house, coded platform for binning, deisotoping, and conversion into the imzML format. The imzML files were then imported to LipostarMSI software [33] for image co-registration, statistical analysis, image visualization, processing, generation, and lipid identification. In particular, the region of interest (ROI) was determined based on the bisecting k-means segmentation analysis [14]. A co-localization algorithm, based on the Pearson correlation between the selected spatial mask and the intensities of an ion image [34], was then applied to isolate the ion markers statistically correlated to specific regions. The ion markers were further confirmed by inspecting the ion images, and their scale intensities were adjusted for the best-overlaid visualization. The elementary composition and the putative identity of the molecules were automatically assigned by LipostarMSI based on accurate mass matching (≤2.5 ppm) and most possible ion forms detected by MALDI referring to the LIPID MAPS Database (http://www.lipidmaps.org/ accessed on 3 June 2019). The lists were then exported by best match scores. The acquired LC–MS data from the serial section homogenates were imported into LipostarMSI to specifically identify the potential markers of the ROIs. The final “identified” molecules from the IMS data were defined as those that could be definitively identified in the LC–MS by the generated fragments. Those that were detected in both IMS and LC–MS data but did not generate sufficient fragments for definitive identification were defined as “annotated”.

## 4. Conclusions

Collectively, in this study, we demonstrated that the combined high resolution molecular and histology imaging planes can capture the physiological process of sebocyte migration in the SG as a part of the holocrine secretion process. In addition, we characterized the molecular basis underlying the heterogeneous layers of the hair follicle bulge region linked to cyclic growth. A number of lipids involved in the dynamic physiology processes of sebaceous glands or hair follicles were specifically annotated and can serve as specific markers, which can be viewed as separate or combined channels. Our current study provides a relatively comprehensive list of SG and hair follicle lipid markers that are mapped to fine features associated with biological processes. These molecules can selectively serve as biomarkers for drug pharmacology or toxicology studies specifically targeting either SGs or follicles. In addition, they can also serve as drug delivery markers for topical delivery studies through these skin appendages. Furthermore, pathway analysis of histology-specific markers can potentially elucidate novel therapeutic targets.

## Data Availability

Datasets related to this article can be found at http://dx.doi.org/10.17632/7mt4gm8wc6.1 (accessed on 14 January 2021), an open-source online data repository hosted at Mendeley Data (Xie, Fang (2021), “Minipig Skin MALDI Imaging Mass Spectrometry”).

## Supplementary Materials

The following supporting information can be downloaded at: https://www.mdpi.com/article/10.3390/ph15040411/s1, Table S1: List of lipids identified or annotated in minipig sebaceous glands, Table S2: List of lipids identified or annotated in minipig epidermis stratum corneum, Table S3: List of lipids identified in minipig hair follicles, Figure S1: Sections collected from minipig skin for MALDI IMS analysis and H&E staining, Figure S2: H&E staining images of S2T3 and S2T5 in comparison with the optical images of S1T3 and S1T5, Figure S3: H&E staining of the three hair follicles identified on the minipig skin sections, Figure S4: Ion images of the same panel of makers displayed in Figure 5 on section S1T3 which is approximately 100 µm away from S1T6, Figure S5: The ion image of *m*/*z* 745.6229 (a hair shaft marker) along with the same panel of makers displayed in Figure 5 on section S1T6 showing hair follicle-1, follicle-2, and follicle-3.
