# Supplementary material for: Mapping the Lipids of Skin Sebaceous Glands and Hair Follicles by High Spatial Resolution MALDI Imaging Mass Spectrometry
Source: Pharmaceuticals (Basel). 2022 Mar 28;15(4):411. doi: 10.3390/ph15040411 (PMC9031257; doi:10.3390/ph15040411)
Supplement: Supplementary file 1 [file pharmaceuticals-15-00411-s001.zip › pharmaceuticals-1610340-supplementary.pdf]

SUPPLEMENTAL DATA FOR

Mapping the Lipids of Skin Sebaceous Glands and Hair Follicles by High Spatial Resolution MALDI Imaging Mass Spectrometry

Fang Xie [1], Mark Reid Groseclose [1], Sara Tortorella [2], Gabriele Cruciani [3] and Stephen Castellino [4,*]

[1] Bioimaging Department, GlaxoSmithKline, Collegeville, PA 19426; fang.x.xie@gsk.com (F.X.); reid.x.groseclose@gsk.com (M.R.G.)
[2] Molecular Horizon srl, Via Montelino 30, 06084, Bettona, Perugia, Italy; sara@molhorizon.it (S.T.)
[3] Department of Chemistry, Biology and Biotechnology, University of Perugia, Via Elce di Sotto 8, 06123, Perugia, Italy; gabriele.cruciani@unipg.it (G.C.)
[4] Xenovista LLC, Chapel Hill, NC, 27516, USA; sxcastellino@xenovista.com (S.C.)
* Correspondence: sxcastellino@xenovista.com

Table S1 List of lipids identified or annotated in minipig sebaceous glands

ROI	Identified; Annotated	Detected m/z	Formula	Lipid Class	Sum Composition	Theoretical Mass [M]	Mass delta (ppm)	Detected Ion Form	Chains	Structure
The CentralZone	Identified	635.5027	C37H72O5	Diacylglycerol	34:0	596.538	2.4	$[M+K]^+$	16:0/18:0/0:0	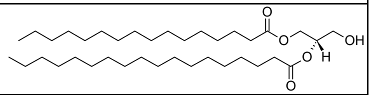
		661.5175	C39H74O5	Diacylglycerol	36:1	622.5536	1.1	$[M+K]^+$	18:0/18:1(9Z)/0:0	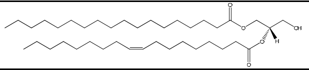
		813.6947	C50H94O6	Triacylglycerol	47:1	790.705	0.5	$[M+Na]^+$	14:0/15:0/18:1(9Z)	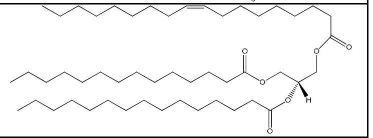
	Annotated	413.3051	C27H42O4	Vitamin D3 and derivative		430.3083	0.3	$[M+H-H_2O]^+$		
		659.5013	C39H72O5	Diacylglycerol	36:2	620.538	0.2	$[M+K]^+$		
		663.5342	C39H76O5	Diacylglycerol	36:0	624.5693	2.6	$[M+K]^+$		
		813.6851	C47H93N2O6P	Ceramide phosphocholine (sphingomyelins)	42:2	812.6771	0.9	$[M+H]^+$		
		815.7095	C50H96O6	Triacylglycerol	47:0	792.7207	0.5	$[M+Na]^+$		
The Peripheral Zone	Identified	716.5235	C39H74NO8P	Diacylglycerophosphocholine	31:2	715.5152	1.4	$[M+H]^+$	14:0/17:2(9Z,12Z)	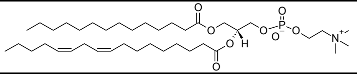
		718.5385	C39H76NO8P	Diacylglycerophosphocholine	31:1	717.5309	0.6	$[M+H]^+$	15:0/16:1(9Z)	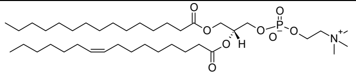
		706.5381	C38H76NO8P	Diacylglycerophosphocholine	30:0	705.5309	0	$[M+H]^+$	14:0/16:0	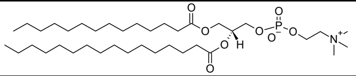
		772.5852	C43H82NO8P	Diacylglycerophosphocholine	35:2	771.5778	0.2	$[M+H]^+$	17:0/18:2(9Z,12Z)	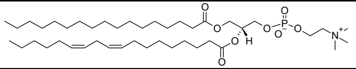
		704.5234	C38H74NO8P	Diacylglycerophosphocholine	30:1	703.5152	1.3	$[M+H]^+$	15:0/15:1(9Z)	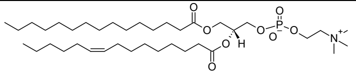

		730.5391	C40H76NO8P	Diacylglycerophosphocholine	32:2	729.5309	1.4	$[M+H]^+$	**	*** 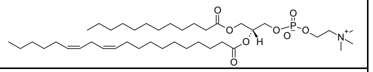
	Annotated	683.4054	C35H65O8P*	Diacylglycerophosphate	32:2	644.4417	0.8	$[M+K]^+$		
		667.4322	C35H65O8P*	Diacylglycerophosphate	32:2	644.4417	1.8	$[M+Na]^+$		
		681.4475	C36H67O8P	Diacylglycerophosphate	33:2	658.4574	1.4	$[M+Na]^+$		
		702.5076	C38H72NO8P*	Diacylglycerophosphocholine	30:2	701.4996	1.1	$[M+H]^+$		
		740.4662	C38H72NO8P*	Diacylglycerophosphocholine	30:2	701.4996	4.7	$[M+K]^+$		

* molecule is detected as more than one ion forms

** multiple possibilities based on available data

*** one structure is listed when there are more than three possibilities

Table S2 Enzymes catalyzing the metabolism of DGs and TGs

Glycerolipid Class	Metabolism	Enzyme Abbreviated in the Pathway Analysis	Enzyme Commision Number (EC Number)	Accepted Name	Alternative Name	Reaction catalysed
Diacylglycerol (DGs)	Formation	MGAT1	2.3.1.22	2-acylglycerol O-acyltransferase	Monoglyceride acyltransferase; Acylglycerol palmitoyltransferase	Acyl-CoA + 2-acylglycerol <=> CoA + diacylglycerol
Diacylglycerol (DGs)	Formation	LPL	3.1.1.34	Lipoprotein lipase	Diacylglycerol lipase; Clearing factor lipase; Diglyceride lipase	Triacylglycerol + H(2)O <=> diacylglycerol + a carboxylate
Diacylglycerol (DGs)	Formation	PAP(Lipin)1-3	3.1.3.4	Phosphatidate phosphatase	Lipin; Phosphatic acid phosphatase; Phosphatic acid phosphohydrolase; Phosphatidic acid phosphatase	A 1,2-diacylglycerol 3-phosphate + H(2)O <=> a 1,2-diacyl-sn-glycerol + phosphate
Diacylglycerol (DGs)	Catabolism	PNPLA2	3.1.1.3	Triacylglycerol lipase	Lipase; Tributyrase; Triglyceride lipase	Triacylglycerol + H(2)O <=> diacylglycerol + a carboxylate<br>Diacylglycerol + H(2)O <=> monoacylglycerol + a carboxylate
Diacylglycerol (DGs)	Catabolism	DGKs	2.7.1.107	Diacylglycerol kinase (ATP)	Diglyceride kinase	ATP + 1,2-diacyl-sn-glycerol <=> ADP + 1,2-diacyl-sn-glycerol 3-phosphate
Triacylglycerol (TGs)	Formation	DGAT1-2	2.3.1.20	Diacylglycerol O-acyltransferase	Diglyceride acyltransferase	Acyl-CoA + 1,2-diacylglycerol <=> CoA + triacylglycerol
Triacylglycerol (TGs)	Catabolism	LPL	3.1.1.34	Lipoprotein lipase	Diacylglycerol lipase; Clearing factor lipase; Diglyceride lipase	Triacylglycerol + H(2)O <=> diacylglycerol + a carboxylate
Triacylglycerol (TGs)	Catabolism	PNPLA2	3.1.1.3	Triacylglycerol lipase	Lipase; Tributyrase; Triglyceride lipase	Triacylglycerol + H(2)O <=> diacylglycerol + a carboxylate<br>Diacylglycerol + H(2)O <=> monoacylglycerol + a carboxylate

Table S3 List of lipids identified or annotated in minipig epidermis stratum corneum

Identified; Annotated	Detected m/z	Formula	Lipid Class	Sum Composition	Theoretical Mass	Mass delta ppm	Detected Ion Form	Chain	Structure
Identified	744.4948	C38H76NO8P	Diacylglycerophosphocholine	30:0	705.5309	1.1	$[M+K]^{+}$	14:0/16:0	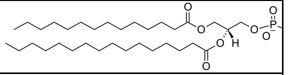
	892.6777	C50H96NO8P	Diacylglycerophosphocholine	42:2	869.6874	1.3	$[M+Na]^{+}$	**	*** 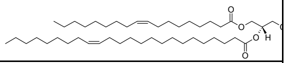
Annotated	705.5543	C38H77N2O7P	Ceramide phosphoethanolamine	36:1	704.5468	0.3	$[M+H]^{+}$		
	745.6229	C42H85N2O6P*	Ceramide phosphoethanolamine	40:1	744.6145	1.5	$[M+H]^{+}$		
	767.6051	C42H85N2O6P	Ceramide phosphoethanolamine	40:1	744.6145	1.7	$[M+Na]^{+}$		
	844.6788	C48H94NO8P*	Diacylglycerophosphocholine	40:1	843.6717	0.2	$[M+H]^{+}$		
	866.6612	C48H94NO8P	Diacylglycerophosphocholine	40:1	843.6717	0.3	$[M+Na]^{+}$		

* molecule is detected as more than one ion forms
** multiple possibilities based on available data
*** one structure is listed when there are more than three possibilities

Table S4 List of lipids identified in minipig hair follicles

ROI	Identified; Annotated	Detected m/z	Formula	Lipid Class	Sum Composition	Theoretical Mass	Mass delta (ppm)	Detected Ion Form	Chain	Structure
ORS	Identified	703.5748	C39H79N2O6P*	Ceramide phosphocholine (sphingomyelin)	34:1	702.5676	0.1	[M+H]$^+$	**	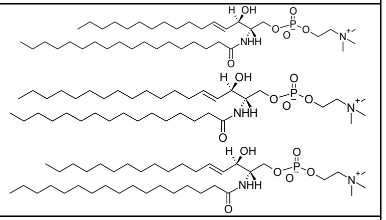
		725.5567	C39H79N2O6P	Ceramide phosphocholine (sphingomyelin)	34:1	702.5676	0.1	[M+Na]$^+$	**	
		734.5695	C40H80NO8P	Diacylglycerophosphocholine	32:0	733.5622	0.1	[M+H]$^+$	16:0/16:0	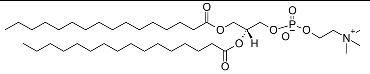
		756.5517	C40H80NO8P*	Diacylglycerophosphocholine	32:0	733.5622	0.4	[M+Na]$^+$	16:0/16:0	
		760.585	C42H82NO8P*	Diacylglycerophosphocholine	34:1	759.5778	0.1	[M+H]$^+$	**	*** 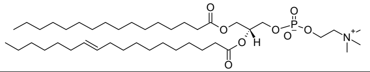
		782.5671	C42H82NO8P	Diacylglycerophosphocholine	34:1	759.5778	0.1	[M+Na]$^+$	**	
		848.5622	C46H84NO8P*	Diacylglycerophosphocholine	38:4	809.5935	0.5	[M+K]$^+$	20:4/18:0	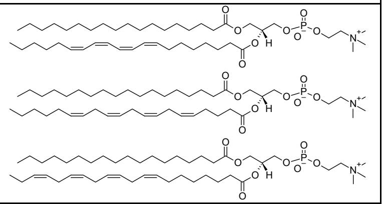
		832.5832	C46H84NO8P	Diacylglycerophosphocholine	38:4	809.5935	0.6	[M+Na]$^+$	20:4/18:0	
		833.6505	C47H91N2O6P	Ceramide phosphocholine (sphingomyelin)	42:3	810.6615	0.2	[M+Na]$^+$	d18:2/24:1	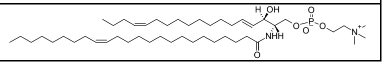

		837.6819	C47H95N2O6P*	Ceramide phosphocholines (sphingomyelin)	42:1	814.6928	0.1	[M+Na]+	**	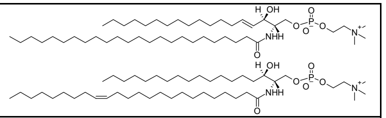
		815.6998	C47H95N2O6P	Ceramide phosphocholines (sphingomyelin)	42:1	814.6928	0.4	[M+H]+	**	
		853.6555	C47H95N2O6P	Ceramide phosphocholines (sphingomyelin)	42:1	814.6928	0.5	[M+K]+	**	
	Annotated	713.4524	C37H71O8P	Diacylglycerophosphate	34:1	674.4887	0.8	[M+K]+		
		739.4668	C39H73O8P	Diacylglycerophosphate	36:2	700.5043	1	[M+K]+		
		737.4519	C39H71O8P*	Diacylglycerophosphate	36:3	698.4887	0.2	[M+K]+		
		721.4781	C39H71O8P	Diacylglycerophosphate	36:3	698.4887	0.3	[M+Na]+		
		741.5209	C40H79O7P	1-alkyl,2-acylglycerophosphate	37:1	702.5563	1.9	[M+K]+		
		772.5251	C43H76NO7P	1-alkyl,2-acylglycerophosphoethanolamine	38:6	749.5359	0.1	[M+Na]+		
		820.5253	C44H80NO8P*	Diacylglycerophosphocholine	36:4	781.5622	0	[M+K]+		
		804.5517	C44H80NO8P	Diacylglycerophosphocholine	36:4	781.5622	0.4	[M+Na]+		
		835.6663	C47H93N2O6P*	Ceramide phosphocholine (sphingomyelin)	42:2	812.6771	0	[M+Na]+		
		851.641	C47H93N2O6P	Ceramide phosphocholine (sphingomyelin)	42:2	812.6771	0.8	[M+K]+		
		813.6851	C47H93N2O6P	Ceramide phosphocholine (sphingomyelins)	42:2	812.6771	0.9	[M+H]+		
IRS	Annotated	737.5101	C40H75O8P	Dialkylglycerophosphoglycerols		714.52	1.3	[M+Na]+		
		796.5842	C45H82NO8P	Diacylglycerophosphocholine	37:4	795.5778	1.1	[M+H]+		
		840.5879	C45H88NO8P	Diacylglycerophosphocholine	37:1	801.6248	0.1	[M+K]+		
Medulla	Annotated	596.5974	C38H77NO3	N-acylsphinganine (dihydroceramide)	38:0	595.5903	0.3	[M+H]+		

		683.4985	C37H73O7P	1-alkyl,2-acylglycerophosphate	34:1	660.5094	0.1	[M+Na]+		

* molecule is detected as more than one ion forms
**multiple possibilities based on available data
*** one structure is listed when there are more than three possibilities

Figure S1. Sections collected from minipig skin for MALDI IMS analysis and H&E staining.

Figure S2. H&E staining images of S2T3 and S2T5 in comparison with the optical images of S1T3 and S1T5.

Figure S3. H&E staining of the three hair follicles identified on the minipig skin sections.

Figure S4. Ion images of the same panel of makers displayed in Figure 5 on section S1T3 which is approximately 100 μm away from S1T6.

Figure S5. The ion image of m/z 705.5543 (a hair shaft marker) along with the same panel of makers displayed in Figure 5 on section of S1T6 showing hair follicle-1, follicle-2, and follicle-3.

Figure S1

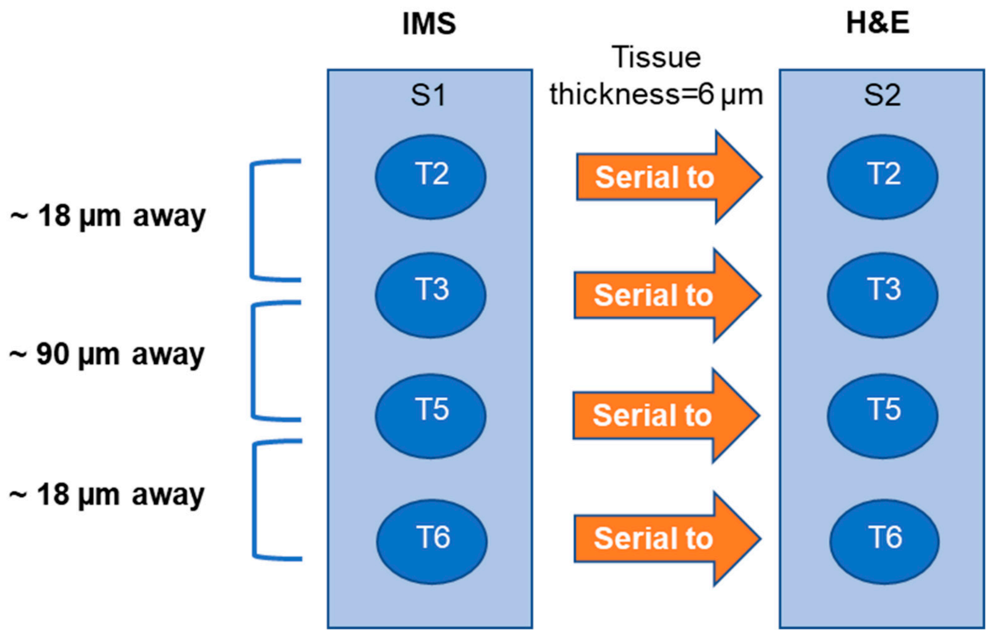

Figure S2

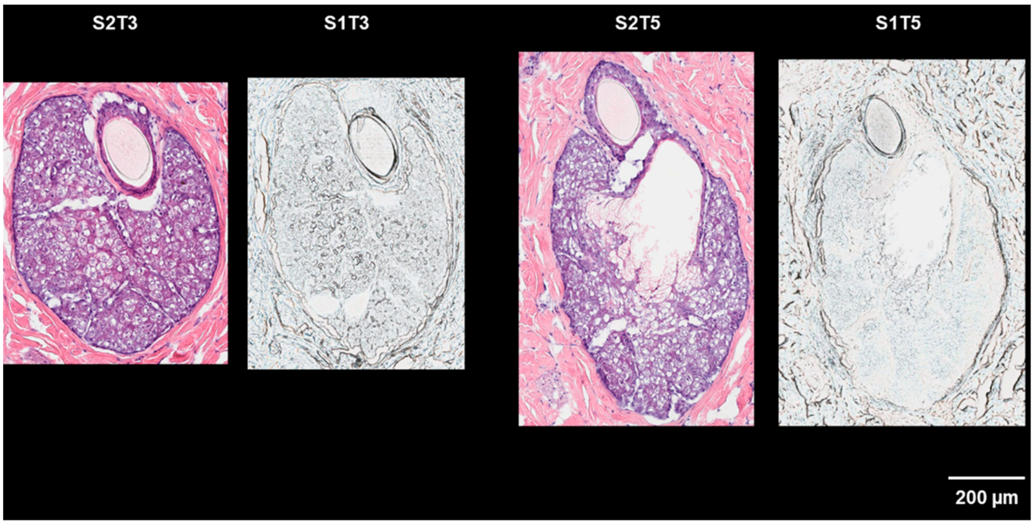

Figure S3

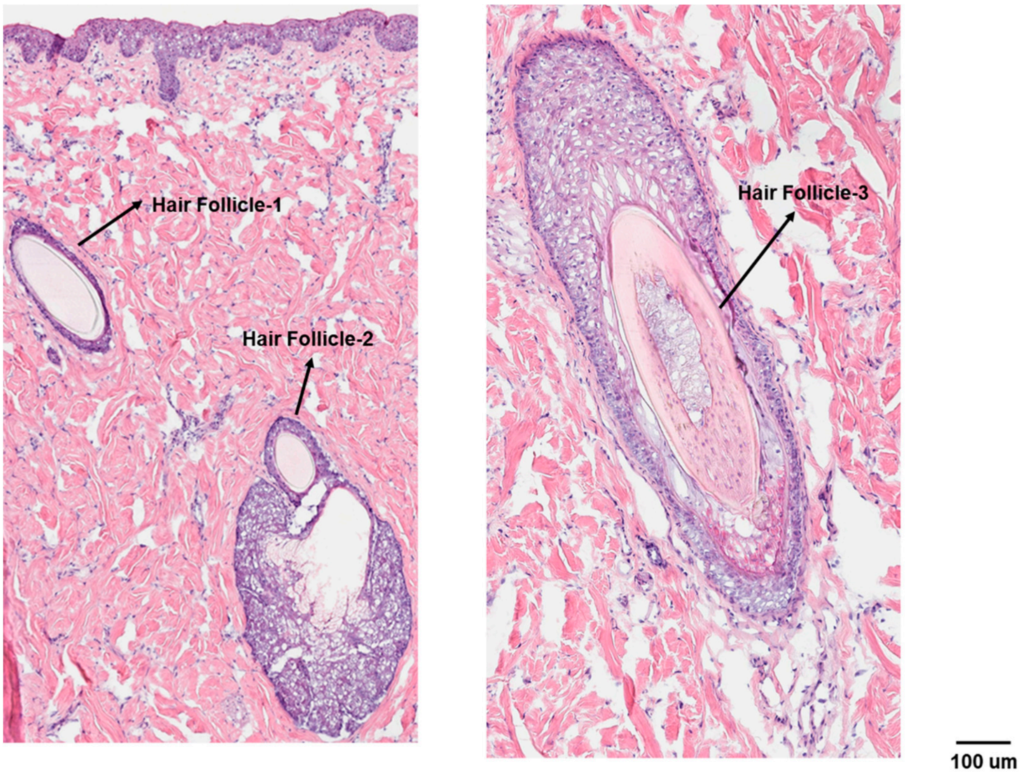

Figure S4

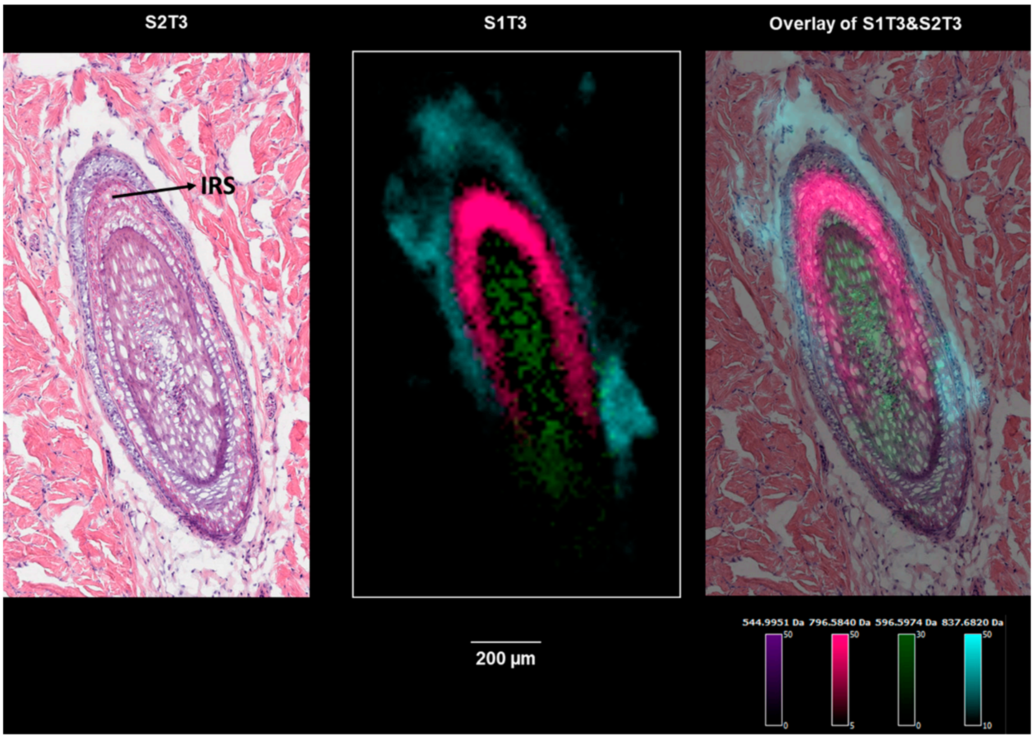

S2T3
S1T3
Overlay of S1T3&S2T3
IRS
200 µm
544.9951 Da
796.5840 Da
596.5974 Da
837.6820 Da
50
0
50
5
30
0
50
10

Figure S5

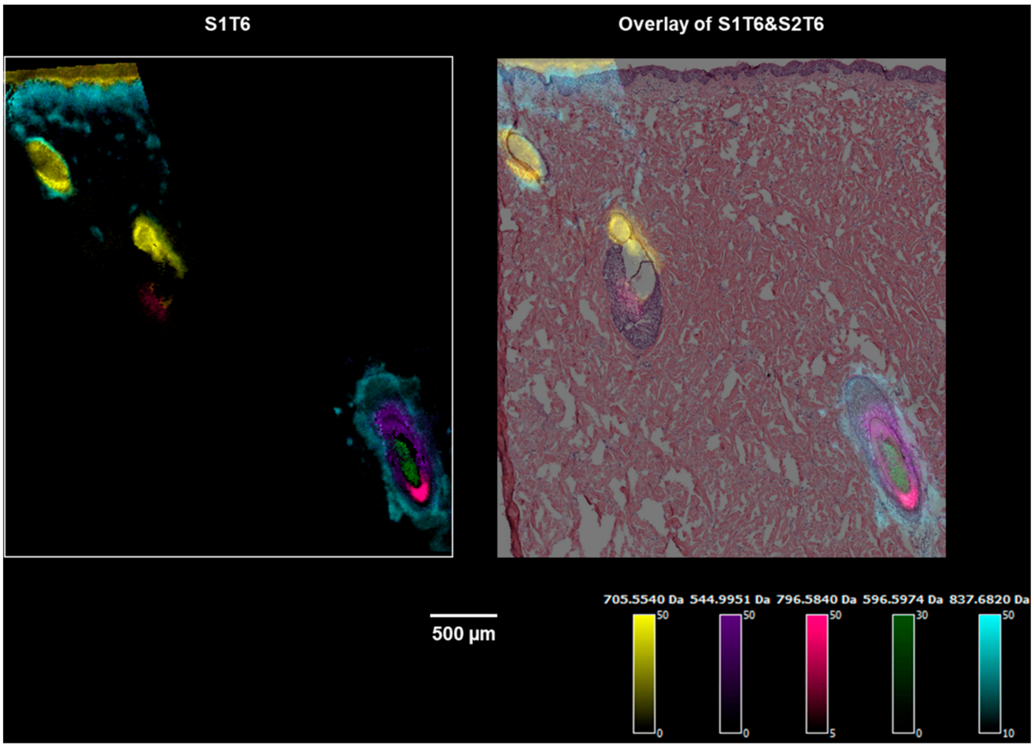
S1T6
Overlay of S1T6&S2T6
500 µm
705.5540 Da
544.9951 Da
796.5840 Da
596.5974 Da
837.6820 Da
50
0
50
0
50
5
30
0
50
10
